# Evaluation of 100 Dutch cases with 16p11.2 deletion and duplication syndromes; from clinical manifestations towards personalized treatment options

**DOI:** 10.1038/s41431-024-01601-2

**Published:** 2024-04-11

**Authors:** Niels Vos, Lotte Kleinendorst, Liselot van der Laan, Jorrit van Uhm, Philip R. Jansen, Agnies M. van Eeghen, Saskia M. Maas, Marcel M.A.M. Mannens, Mieke M. van Haelst

**Affiliations:** 1 Amsterdam UMC, University of Amsterdam, Department of Human Genetics, Meibergdreef 9, Amsterdam, The Netherlands; 2 Amsterdam Reproduction and Development research institute, Amsterdam, The Netherlands; 3 Amsterdam UMC, University of Amsterdam, Emma Center for Personalized Medicine, Meibergdreef 9, Amsterdam, The Netherlands; 4 Department of Complex Trait Genetics, Center for Neurogenomics and Cognitive Research, VU University, Amsterdam, the Netherlands; 5 Emma Children’s Hospital, University of Amsterdam, Amsterdam, The Netherlands

**Keywords:** Genetics research, Chromosome abnormality, Cytogenetics, Body mass index

## Abstract

The 16p11.2 deletion syndrome is a clinically heterogeneous disorder, characterized by developmental delay, intellectual disability, hyperphagia, obesity, macrocephaly and psychiatric problems. Cases with 16p11.2 duplication syndrome have similar neurodevelopmental problems, but typically show a partial ‘mirror phenotype’ with underweight and microcephaly. Various copy number variants (CNVs) of the chromosomal 16p11.2 region have been described. Most is known about the ‘typical’ 16p11.2 BP4-BP5 (29.6–30.2 Mb; ~600 kb) deletions and duplications, but there are also several published cohorts with more distal 16p11.2 BP2-BP3 CNVs (28.8–29.0 Mb; ~220 kb), who exhibit clinical overlap. We assessed 100 cases with various pathogenic 16p11.2 CNVs and compared their clinical characteristics to provide more clear genotype-phenotype correlations and raise awareness of the different 16p11.2 CNVs. Neurodevelopmental and weight issues were reported in the majority of cases. Cases with distal 16p11.2 BP2-BP3 deletion showed the most severe obesity phenotype (73.7% obesity, mean BMI SDS 3.2). In addition to the more well defined typical 16p11.2 BP4-BP5 and distal 16p11.2 BP2-BP3 CNVs, we describe the clinical features of five cases with other, overlapping, 16p11.2 CNVs in more detail. Interestingly, four cases had a second genetic diagnosis and 18 cases an additional gene variant of uncertain significance, that could potentially help explain the cases’ phenotypes. In conclusion, we provide an overview of our Dutch cohort of cases with various pathogenic 16p11.2 CNVs and relevant second genetic findings, that can aid in adequately recognizing, diagnosing and counseling of individuals with 16p11.2 CNVs, and describe the personalized medicine for cases with these conditions.

## Introduction

The human chromosome 16p11.2 region consists of low copy repeats, that are susceptible to misalignment during recombination, resulting in non-allelic homologous recombination [1] and leading to recurrent CNVs at different breakpoints (BPs). Recurrent 16p11.2 deletions and duplications are amongst the most prevalent disease-causing and development-affecting chromosomal copy number variations (CNVs) [2]. Although clinically heterogeneous, they share clinical features such as developmental delay (DD), intellectual disability (ID), behavioral and psychiatric problems, weight issues, congenital anomalies and epilepsy [3].

The ‘typical’ 16p11.2 BP4-BP5 deletion (OMIM#611913) and duplication (OMIM#614671) affect the ~600 kilobases (kb) ~29.6 to 30.2 Megabases (Mb) region of chromosome 16, while the ‘distal’ 16p11.2 BP2-BP3 CNVs (OMIM#613444) comprise the ~220 kb ~28.8–29 Mb region of chromosome 16 (reference genome GRCh37/hg19). Studies have shown that cases with a typical 16p11.2 BP4-BP5 CNV on average have a 22-26 points lower full scale intelligence quotient (FSIQ) score than relatives without the CNV [4–6]. Similarly, cases with a distal 16p11.2 BP2-BP3 CNV on average have a lower FSIQ and more often neurodevelopmental disorders than non-carrier relatives [7]. Penetrance of distal 16p11.2 BP2-BP3 deletions for neurodevelopmental disorders, however, is believed to be lower than the penetrance of typical 16p11.2 BP4-BP5 deletions [8]. Autism spectrum disorder (ASD) is reported in ~25% of cases with 16p11.2 BP2-BP3 and BP4-BP5 CNVs [4, 9, 10]. Interestingly, some opposite symptoms (‘mirror phenotypes’), have been described in cases with deletions compared to those with duplications. Cases with typical 16p11.2 BP4-BP5 deletion or distal 16p11.2 BP2-BP3 deletion have an increased risk of developing obesity and macrocephaly, while cases with a typical 16p11.2 BP4-BP5 duplication or a distal 16p11.2 BP2-BP3 duplication more often present with underweight and microcephaly [10, 11]. Studies so far have mainly focused on the typical 16p11.2 BP4-BP5 CNV. Less is known about the phenotypes of cases with distal 16p11.2 BP2-BP3 CNVs.

The typical 16p11.2 BP4-BP5 deletion has an estimated prevalence of 1:2000, typical 16p11.2 BP4-BP5 duplication 1:2500, distal 16p11.2 BP2-BP3 deletion 1:4100 and distal 16p11.2 BP2-BP3 duplication 1:1500, suggesting an estimated combined prevalence of these pathogenic recurrent typical 16p11.2 BP4-BP5 and distal 16p11.2 BP2-BP3 CNVs in the general population of 1 in 600 [2, 12]. In clinical cohorts, these CNVs have a higher prevalence (e.g. distal 16p11.2 BP2-BP3 deletion 1:400-1000, depending on the clinical cohort) [13–17]. Surprisingly, many clinicians, are not familiar with 16p11.2 CNVs and their potential clinical consequences, which hampers a timely genetic diagnosis and adequate guidance and treatment for the majority of these cases. Parents with subtle symptoms can carry the same CNV as their more severely affected child. Typical 16p11.2 BP4-BP5 duplications occur de novo in ~25% and typical 16p11.2 BP4-BP5 deletions in ~93% [18, 19]. Information of parental and environmental factors that could affect eventual phenotypes is (still) lacking in the existing literature.

We here provide a detailed description of our Dutch cohort of 100 cases with pathogenic 16p11.2 CNVs, in order to better understand the clinical consequences of these CNVs. Moreover, the genetic subgroups are studied to delineate genotype-phenotype correlations, which helps further development of syndrome-specific clinical guidelines and improve consultation and personalized medicine options for cases with 16p11.2 CNVs.

## Subjects and methods

### Participants

The cohort consists of children and adults with pathogenic 16p11.2 region affecting CNVs, who visited our genetics clinic at Amsterdam University Medical Centers between 2017 and 2022.

### Medical ethical statement

Verbal and written consent was obtained from patients and/or their families.

### Clinical information

We used patient- and/or parent-reported medical history and information from medical files. Detailed phenotyping (medical and family history, body measurements, dysmorphological assessment) was performed by experienced clinical geneticists.

Birthweight centiles for gestational age and sex were defined by the Perined database [20]. Small-for-gestational-age (SGA) was defined as birthweight <10th centile, large-for-gestational-age (LGA) as birthweight >90th centile. Dutch national growth charts were used to calculate standard deviation scores (SDS) for occipital frontal circumference (OFC), height, weight-for-height and BMI [21]. Short stature was defined as height-for-age ≤-2 SDS [22] and tall stature as height-for-age ≥2 SDS [23]. For children 0-2 years, weight-for-age and weight-for-length curves were used to determine weight status and weight-for-length <-2 SDS was considered underweight and >2 SDS overweight. For children 2–19 years old, age- and sex-specific BMI cut-offs [24] were used to determine weight status. For adults, these BMI cut-offs (kg/m²) were used: <18.5 (underweight), 18.5-25 (normal weight), 25-30 (overweight) and ≥30 (obesity). Microcephaly was defined as OFC ≤ -2 SDS and macrocephaly as OFC ≥ 2 SDS [25]. Intellectual disability (ID) was defined as FSIQ ≤ 70, borderline intellectual functioning as FSIQ 71-85.

### Statistical analysis

R software (version 4.2.3; R Foundation for Statistical Computing, Vienna, Austria) was used for statistical assessment of our cohort. Descriptive statistics were used for frequencies, percentages, means and standard deviations (SD) for normally distributed data, medians, and interquartile range (IQR) for skewed data, minimum and maximum values.

To determine distribution, Kolmogorov-Smirnov and Shapiro-Wilk tests were performed. For the comparison of the means of normally distributed continuous data in two independent groups, we used an independent samples *T* test. For the comparison of groups with non-normally distributed continuous data (e.g., age), we used a Mann–Whitney U or Kruskal-Wallis test. Pearson’s Chi Squared test was used to compare two categorical variables. When necessary, Yate’s correction was applied to the Chi-Squared test. *P* values < 0.05 were considered statistically significant. One case (case 18) was excluded from group analyses, as the second diagnosis could affect the results.

### Diagnostic techniques used

Genetic analyses were performed in different ISO15189-certified diagnostic laboratories in the Netherlands and in Centrum Medische Genetica Universitair Ziekenhuis Antwerpen (Belgium). Genomic DNA was analyzed with different chromosomal microarray (CMA) methods, such as Array 180k comparative genomic hybridization (CGH) and Single Nucleotide Polymorphism (SNP) Array according to the instruction of the supplier in different years (2009–2022). Genomic locations mentioned in this article are based on NCBI Build (GRCh37/hg19).

### Genetic test results

Genetic testing was initially performed for various clinical reasons, such as DD/learning difficulties, obesity and/or epilepsy. Additional genetic analysis for family members was performed if parents and relatives consented to do so and were referred to us. Relatives with less (severe) symptoms, yet the same 16p11.2 CNV, were also asked for permission to use their medical information in our database. Additional genetic testing (karyotyping, Fragile X diagnostics, Next Generation Sequencing; NGS, Whole Exome Sequencing; WES, and/or genome-wide DNA methylation analysis; EpiSign) was performed prior to 16p11.2 CNV diagnosis and in cases with severe/remarkable symptoms that could indicate a second genetic diagnosis (e.g. remarkable short stature, dysmorphic features, severe DD or lower than expected IQ).

## Results

### Baseline characteristics

A total of 100 cases with pathogenic 16p11.2 CNVs were included. Male/female ratio was 52/48. Median age was 10.7 years (IQR 6.9–17.3) [range 0.6–60.5 years]. The majority of cases (76%) was <18 years.

### Genetic subgroups

**Fig. 1:**
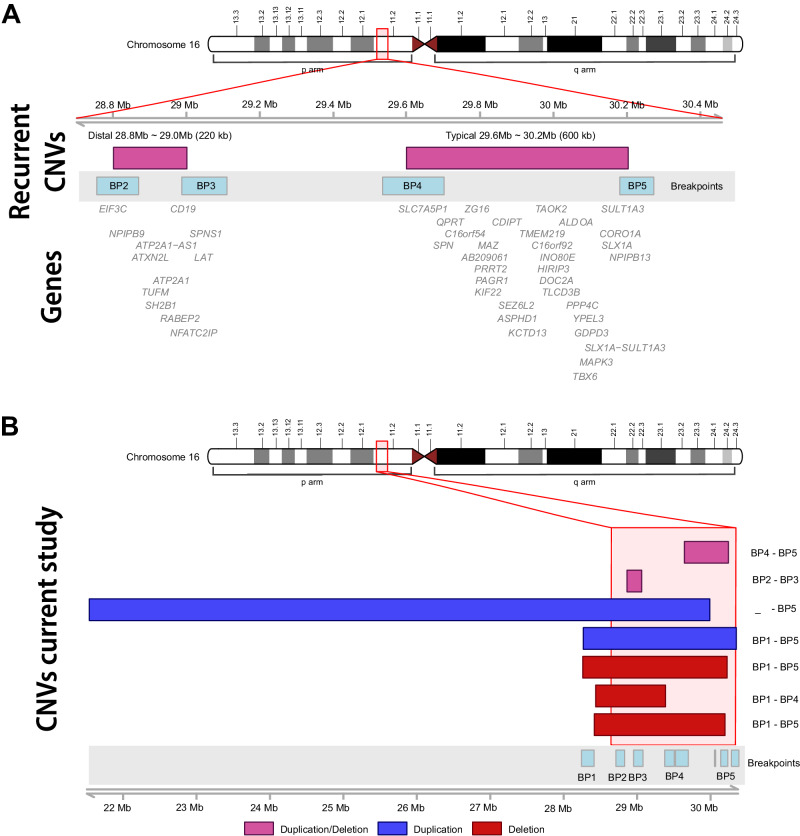
Overview of different 16p11.2 CNVs in our cohort. This figure provides an overview of chromosome 16, including its short (p) and long (q) arms, as well as chromosomal bands and 16p11.2 breakpoints. **A** The recurrent typical ~600 kb 16p11.2 BP4-BP5 and distal ~220 kb 16p11.2 BP2-BP3 CNVs (deletions and duplications) are shown in more detail, including breakpoints (BPs) and genes in these regions. **B** The different 16p11.2 CNVs in our cohort, including the larger ‘other’ groups that are included in this study are shown, including the breakpoints.

**Fig. 2:**
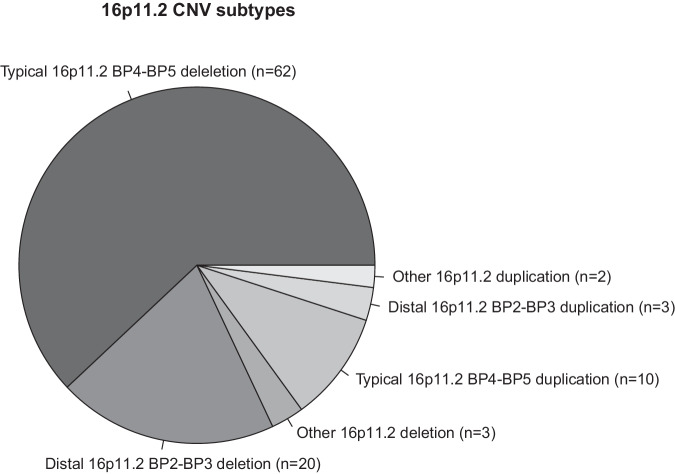
Frequencies of 16p11.2 CNV subtypes in our cohort. Depicted are the different 16p11.2 CNVs identified in our cases, mainly consisting of typical 16p11.2 BP4-BP5 and distal 16p11.2 BP2-BP3 deletions (del) and duplications (dup), but also other (larger) deletions and duplications.

Typical 16p11.2 BP4-BP5 deletion (*n* = 62), distal 16p11.2 BP2-BP3 deletion (*n* = 20), typical 16p11.2 BP4-BP5 duplication (*n* = 10) and distal 16p11.2 BP2-BP3 duplication (*n* = 3) were most frequently reported. In addition, five other (larger and partially overlapping) 16p11.2 CNVs were labeled as ‘other’ 16p11.2 deletions and duplications (Table 1, Figs. 1–2).

**Table 1:** Overview of the 16p11.2 CNVs in our cohort, the chromosomal regions (reference genome GRCh37/hg19), CNV sizes and categories.

Del/dup	Region (Mb)	Size	Category	*N*
Del	29.6–30.2	600 kb	Typical BP4-BP5	62
Del	28.8–29	220 kb	Distal BP2-BP3	20
Del	28.3–30.2	1.9 Mb	Other	1
Del	28.4–30.2	1.8 Mb	Other	1
Del	28.4–29.4	1 Mb	Other	1
Dup	29.6–30.2	600 kb	Typical BP4-BP5	10
Dup	28.8–29	220 kb	Distal BP2-BP3	3
Dup	21.5–30	8.5 Mb	Other	1
Dup	28.3–30.3	2 Mb	Other	1

*Del* deletion, *dup* duplication.

### Second confirmed genetic diagnosis

Additional genetic analyses, other than CMA, were performed in 58% of the total cohort (see “Methods”). A second confirmed genetic diagnosis was found in 4% of the total cohort (Table 2). A variant of uncertain significance (another CNV or a single gene variant) was reported in 18%.

**Table. 2:** Confirmed second genetic diagnoses in four cases with 16p11.2 deletion, the involved genes and number (#) in the Online Mendelian Inheritance in Man (OMIM) database https://www.omim.org/.

Case	16p11.2 CNV	Second diagnosis	Gene	OMIM #	Variant	Classification	Cytogenic location	Reason additional genetic testing
#11	Typical BP4-BP5 deletion	Cleft palate with ankyloglossia	*TBX22*	303400	NM_001109878.1: c.1310del p.(Pro437Leufs*49), hemizygous	Likely pathogenic (class 4)	Xq21.1	Cleft palate, hydrocephalus
#17	Typical BP4-BP5 deletion	Immunodeficiency	*PIK3CD*	615513	NM_005026.4: c.3061 G > A p.(Glu1021Lys), heterozygous	Pathogenic (class 5)	1p36.22	Common Variable Immune Deficiency (CVID)
#18	Distal BP2-BP3 deletion	Noonan syndrome	*PTPN11*	163950	NM_002834.3: c.922 A > G p.(Asn308Asp), heterozygous	Pathogenic (class 5)	12q24.13	Short stature, heart defect
#22	Distal BP2-BP3 deletion	Cystic fibrosis	*CFTR*	219700	NM_000492.3: c.1521_1523del p.(Phe508del) NM_000492.4: c.350 G > A p.(Arg117His)	Pathogenic (class 5) / Pathogenic (class 5)	7q31.2	Respiratory insufficiency, recurrent pneumonia

### Clinical features

**Fig. 3:**
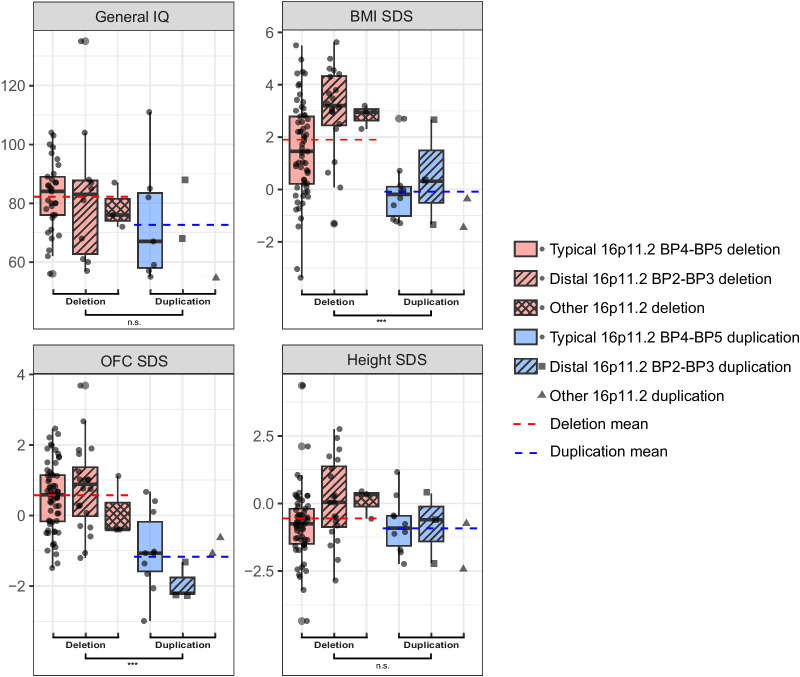
Comparison of FSIQ and growth between 16p11.2 deletion and duplication subgroups. Comparative boxplots representing General IQ, BMI SDS, OFC SDS, and height SDS across 16p11.2 deletion (red) and –duplication (blue) subgroups. Mean values for the deletion and duplication groups are illustrated with blue and red horizontal lines, respectively. A *t* test was performed to identify statistically significant differences between the deletion and duplication groups, * (*p* < 0.05), ** (*p* < 0.01), and *** (*p* < 0.001).

The most common clinical features in our cohort are shown and compared to previously described clinical cohorts in Table 3A, B (16p11.2 deletions) and Table 4 (16p11.2 duplications). Distribution and means of FSIQ, BMI SDS, OFC SDS and height SDS per 16p11.2 CNV subgroup are depicted in Fig. 3. Less common features in our cohort are presented in Supplementary Table S1.

**Table 3:** A. Overview of clinical features of cases with typical 16p11.2 BP4-BP5 deletion and distal 16p11.2 BP2-BP3 deletion in our cohort and comparison of the prevalence of these clinical features to the prevalence in previously published cohorts. B. Comparison of clinical features of cases with typical 16p11.2 BP4-BP5 deletion to clinical features of cases with distal 16p11.2 BP2-BP3 deletion in our cohort.

		Typical 16p11.2 BP4-BP5 deletion							Distal 16p11.2 BP2-BP3 deletion			
		Current study	Literature						Current study	Literature		
Clinical characteristics		Our cohort (total *n* = 62)	Simons Searchlight registry 2023 (PMID: 22445335) (*n* = 222)	Genereviews (updated 2021) (PMID: 20301775)	Niarchou et al. 2019 (PMID: 30664628) (*n* = 217)	D’Angelo et al. 2016 (PMID: 26629640) (n = 390)	Steinman et al. 2016 (PMID: 27410714) (n = 136)	Zufferey et al. 2012 (PMID: 23054248) (*n* = 285)	Our cohort (*n* = 19)	Simons Searchlight registry 2023 (PMID: 22445335) (*n* = 21)	Hanssen et al. 2023, UK Biobank (PMID: 37586323) (*n* = 59)	Loviglio 2017 (PMID: 27240531) (*n* = 88)
General characteristics												
Age (years)	*Median*	11.9							14.6			
	*(IQR)*	(6.9–17.3)							(8.4–33.6)			
	*[Min-Max]*	[1-46.7]							[0.9–60.5]			
Male / Female	*n / n*	30/32							9/10			
Inheritance												
Maternal/Paternal/De novo/Unknown	*n / n / n / n*	7/5/35/15							0/6/2/11			
De novo	*n (%)*	35/47 (74.5)		93%	65%	45%	54%	63%	2/8 (25)			
Pregnancy and neonatal period												
Preterm birth	*n (%)*	7/58 (12.1)						11%	–			
Postterm birth	*n (%)*	6/58 (10.3)							4/19 (21.1)			
SGA	*n (%)*	13/55 (23.6)							2/18 (11.1)			
LGA	*n (%)*	4/55 (7.3)							1/18 (5.6)			
Neonatal feeding problems	*n (%)*	21/62 (33.9)							1/19 (5.3)			
Hypotonia	*n (%)*	4/33 (12.1)	59%				53%		4/10 (40)	48%		
Development and behavior												
DD or ID		≥85.5%	63%	Most (if not all)					≥78.9%	52%		
DD	*n (%)*	53/62 (85.5)		Most (if not all)					15/19 (78.9)			
Speech delay	*n (%)*	43/53 (81.1)	68%	80–90%				83%	11/15 (73.3)	52%		
Motor delay	*n (%)*	23/53 (43.4)		–				38%	13/15 (86.7)			
ID	*n (%)*	6/33 (18.2)		Most not	30%			20%	4/10 (40)			
FSIQ	*Mean (SD)*	84 (11.8)		82.7			84 (16)	76.1 (16.4)	83 (23.8)			
ASD	*n (%)*	12/62 (19.4)	45%	20–25%	22%	16%		11%	4/18 (22.2)	19%	0%	26%
Autistic features or ASD	*n (%)*	34/62 (54.8)							9/17 (52.9)			
ADHD	*n (%)*	9/33 (27.3)	29%	35%	29%				3/4 (75)	33%		
Growth and weight												
Short stature	*n (%)*	8/61 (13.1)							1/19 (5.3)			
Tall stature	*n (%)*	2/61 (3.3)							3/19 (15.8)			
Height SDS	*Mean (SD)*	-0.8 (1.3)							0.1 (1.5)			
Underweight	*n (%)*	2/61 (3.3)							-			
Normal weight	*n (%)*	28/61 (45.9)							3/19 (15.8)			
Overweight	*n (%)*	13/61 (21.3)							2/19 (10.5)			
Obesity^a^	*n (%)*	18/61 (29.5)		75%				75%	14/19 (73.7)			
Age of onset obesity (years)	*Mean (SD)*	9.8 (8.4)							5.1 (4.1)			
BMI SDS	*Mean (SD)*	1.5 (1.8)				~1.3			3.2 (1.7)			~1.3
Hyperphagia	*n (%)*	25/61 (41)							11/18 (61.1)			
Microcephaly	*n (%)*	–	2%				5%		–	5%		
Macrocephaly	*n (%)*	5/61 (8.2)	28%	17%			17%	17%	2/19 (10.5)	10%		
OFC SDS	*Mean (SD)*	0.5 (1)				~0.8	1.4 (1.3)	0.6	0.9 (1.2)			~0.5
Neurological features												
Epilepsy	*n (%)*	6/62 (9.7)	23%	25%		19%	27%	24%	3/19 (15.8)	14%		
Headaches	*n (%)*	12/13 (92.3)							4/5 (80)			
Migraines	*n (%)*	5/6 (83.3)							1/4 (25)			
Sleep problems	*n (%)*	20/62 (32.3)							11/19 (57.9)			
Melatonin use^a^	*n (%)*	8/62 (12.9)							4/19 (21.1)			
Sensory system												
Hearing problems	*n (%)*	3/62 (4.8)		11%		9%			-			
Vision problems	*n (%)*	29/62 (46.8)				14%			12/19 (63.2)			
Olfactory problems	*n (%)*	2/62 (3.2)							-			
Other features												
Scoliosis	*n (%)*	5/62 (8.1)				6%		20%	-			
Cardiovascular problems^b^	*n (%)*	6/62 (9.7)		6%		5%			1/19 (5.3)			
Constipation	*n (%)*	8/58 (13.8)	26%						4/18 (22.2)	43%		
Urogenital problems	*n (%)*	11/18 (61.1)				6%			5/8 (62.5)			
Frequent infections	*n (%)*	32/62 (51.6)							11/18 (61.1)			

Clinical characteristics		Typical 16p11.2 BP4-BP5 del (*n* = 62)	Distal 16p11.2 BP2-BP3 del (*n* = 19)	Typical 16p11.2 BP4-BP5 versus distal 16p11.2 BP2-BP3 del (*p* value)
General characteristics				
Age (years)	*Median (IQR)*	11.9	14.6	0.26
	*[Min-Max]*	(6.9–17.3)	(8.4–33.6)	
		[1–46.7]	[0.9–60.5]	
Male / Female	*n / n*	30/32	9/10	1
Inheritance				
Maternal/Paternal/De novo/Unknown	*n / n / n / n*	7/5/35/15	0/6/2/11	N/A
De novo	*n (%)*	35/47 (74.5)	2/8 (25)	**0.018**
Pregnancy and neonatal period				
Preterm birth	*n (%)*	7/58 (12.1)	0/19 (0)	0.259
Postterm birth	*n (%)*	6/58 (10.3)	4/19 (21.1)	0.417
SGA	*n (%)*	13/55 (23.6)	2/18 (11.1)	0.141
LGA	*n (%)*	4/55 (7.3)	1/18 (5.6)	0.224
Neonatal feeding problems	*n (%)*	21/62 (33.9)	1/19 (5.3)	**0.031**
Hypotonia	*n (%)*	4/33 (12.1)	4/10 (40)	0.128
Development and behavior				
DD	*n (%)*	53/62 (85.5)	15/19 (78.9)	0.748
Speech delay	*n (%)*	43/53 (81.1)	11/15 (73.3)	0.751
Motor delay	*n (%)*	23/53 (43.4)	13/15 (86.7)	**0.010**
ID	*n (%)*	6/33 (18.2)	4/10 (40)	0.358
FSIQ	*Mean (SD)*	84 (11.8)	83 (23.8)	0.992
ASD	*n (%)*	12/62 (19.4)	4/18 (22.2)	1
Autistic features or ASD	*n (%)*	34/53 (64.2)	9/17 (52.9)	0.424
ADHD	*n (%)*	9/33 (27.3)	3/4 (75)	0.174
Growth and weight				
Short stature	*n (%)*	8/61 (13.1)	1/19 (5.3)	0.596
Tall stature	*n (%)*	2/61 (3.3)	3/19 (15.8)	0.154
Height SDS	*Mean (SD)*	-0.8 (1.3)	0.1 (1.5)	**0.007**
Underweight	*n (%)*	2/61 (3.3)	0/19 (0)	1
Normal weight	*n (%)*	28/61 (45.9)	3/19 (15.8)	**0.037**
Overweight	*n (%)*	13/61 (21.3)	2/19 (10.5)	0.474
Obesity^a^	*n (%)*	18/61 (29.5)	14/19 (73.7)	**0.001**
Age of onset obesity (years)	*Mean (SD)*	9.8 (8.4)	5.1 (4.1)	**0.044**
BMI SDS	*Mean (SD)*	1.5 (1.8)	3.2 (1.7)	**0.0001**
Hyperphagia	*n (%)*	25/61 (41)	11/18 (61.1)	0.216
Microcephaly	*n (%)*	–	–	–
Macrocephaly	*n (%)*	5/61 (8.2)	2/19 (10.5)	1
OFC SDS	*Mean (SD)*	0.5 (1)	0.9 (1.2)	0.228
Neurological features				
Epilepsy	*n (%)*	6/62 (9.7)	3/19 (15.8)	0.746
Headaches	*n (%)*	12/13 (92.3)	4/5 (80)	0.218
Migraines	*n (%)*	5/6 (83.3)	1/4 (25)	0.153
Sleep problems	*n (%)*	20/62 (32.3)	11/19 (57.9)	0.08
Melatonin use^a^	*n (%)*	8/62 (12.9)	4/19 (21.1)	0.744
Sensory system				
Hearing problems	*n (%)*	3/62 (4.8)	0/18 (0)	0.777
Vision problems	*n (%)*	29/62 (46.8)	12/19 (63.2)	0.323
Olfactory problems	*n (%)*	2/62 (3.2)	0/18 (0)	1
Other features				
Scoliosis	*n (%)*	5/62 (8.1)	0/12 (0)	0.463
Cardiovascular problems^b^	*n (%)*	6/62 (9.7)	1/19 (5.3)	0.849
Constipation	*n (%)*	8/58 (13.8)	4/18 (22.2)	0.973
Urogenital problems	*n (%)*	11/18 (61.1)	5/8 (62.5)	0.278
Frequent infections	*n (%)*	32/62 (51.6)	11/18 (61.1)	0.828

Bold values indicate statistical significance *p* < 0.05.

*Del* deletion, *SGA* small for gestational age, *LGA* large for gestational age, *DD* developmental delay, *ID* intellectual disability, *FSIQ* full scale intelligence quotient, *ASD* autism spectrum disorder, *ADHD* attention deficit hyperactive disorder.

^a^Currently or previously.

^b^Includes congenital heart defects and later-onset cardiac problems (Supplementary Table S1).

**Table 4:** Overview of clinical features of cases with typical 16p11.2 BP4-BP5 duplication and distal 16p11.2 BP2-BP3 duplication in our cohort and comparison of the prevalence of these clinical features to the prevalence in previously published cohorts.

		Typical 16p11.2 BP4-BP5 duplication					Distal 16p11.2 BP2-BP3 duplication		
		Current study	Literature				Current study	Literature	
Clinical characteristics		Current cohort (*n* = 10)	Simons Searchlight registry 2023 (PMID: 22445335) (*n* = 142)	Niarchou 2019 (PMID: 30664628) (*n* = 114)	D’Angelo 2016 (PMID: 26629640), (*n* = 270)	Steinman et al 2016 (PMID: 27410714) (*n* = 110)	Current cohort (*n* = 3)	Simons Searchlight registry 2023 (PMID: 22445335) (*n* = 20)	Loviglio 2017 (PMID: 27240531) (*n* = 49)
General characteristics									
Age (years)	*Median (IQR)*	8.2					6.4		
	*[Min-Max]*	(5.5–18.7)					(N/A)		
		[1.8–44.7]					[0.6–8.8]		
Male / Female	*n / n*	7/3					2/1		
Inheritance									
Maternal/Paternal/ De novo/Unknown	*n / n / n / n*	4/1/0/5					1/1/0/1		
De novo	*n (%)*	-		19%	17%	12%	–		
Pregnancy and neonatal period									
Preterm birth	*n (%)*	3/9 (33.3)					–		
Postterm birth	*n (%)*	1/9 (11.1)					–		
SGA	*n (%)*	1/8 (12.5)					–		
LGA	*n (%)*	2/8 (25)					–		
Neonatal feeding problems	*n (%)*	3/10 (30)					–		
Hypotonia	*n (%)*	–	46%			42%	–	20%	
Development and behavior									
DD/ID		≥60%	48%				100%	40%	
DD	*n (%)*	6/10 (60)					3/3 (100)		
Speech delay	*n (%)*	4/6 (66.7)	49%				1/2 (50)	55%	
Motor delay	*n (%)*	5/6 (83.3)					1/2 (50)		
ID	*n (%)*	4/7 (57.1)			30.5%		1/2 (50)		
FSIQ	*Mean (SD)*	73.7 (20.3)			78.8	86 (22)	78 (14.1)		
ASD	*n (%)*	1/10 (10)	40%		14%		–	15%	22%
Autistic features or ASD	*n (%)*	6/10 (60)					–		
ADHD	*n (%)*	3/10 (30)	32%				–	25%	
Growth and weight									
Short stature	*n (%)*	1/10 (10)					1/3 (33.3)		
Tall stature	*n (%)*	–					–		
Height SDS	*Mean (SD)*	-0.8 (1)					-0.8 (1.3)		
Underweight	*n (%)*	–					-		
Normal weight	*n (%)*	9/10 (90)					2/3 (66.7)		
Overweight	*n (%)*	–					1/3 (33.3)		
Obesity	*n (%)*	1/10 (10)					–		
BMI SDS	*Mean (SD)*	–0.1 (1.2)			~0		0.6 (2)		~-0.9
Hyperphagia	*n (%)*	1/10 (10)					–		
Microcephaly	*n (%)*	2/10 (20)	15%			17%	2/3 (66.7)	15%	
Macrocephaly	*n (%)*	–	4%			3%	–	-	
OFC SDS	*Mean (SD)*	–1 (1.1)			~-1	-0.3 (1.4)	-1.9 (0.5)		~-1.4
Neurological features									
Epilepsy	*n (%)*	1/10 (10)	14%		14%	29%	–	5%	
Headaches	*n (%)*	–					–		
Scoliosis	*n (%)*	–			8%		–		
Sleep problems	*n (%)*	6/10 (60)					–		
Melatonin use^a^	*n (%)*	4/10 (40)					–		
Sensory system									
Hearing problems	*n (%)*	–			4%		–		
Vision problems	*n (%)*	3/10 (30)			17%		2/3 (66.7)		
Olfactory problems	*n (%)*	–					–		
Other features									
Cardiovascular problems	*n (%)*	–			4%		–		
Constipation	*n (%)*	3/10 (30)	54%				–	30%	
Urogenital problems	*n (%)*	1/10 (10)			8%		1/3 (33.3)		
Frequent infections	*n (%)*	3/10 (30)					2/3 (66.7)		

*Dup* duplication, *SGA* Small for Gestational Age, *LGA* Large for Gestational Age, *DD* developmental delay, *ID* Intellectual Disability, *FSIQ* Full Scale Intelligence Quotient, *ASD* Autism Spectrum Disorder, *ADHD* Attention Deficit Hyperactive Disorder.

^a^Currently or previously.

#### Typical 16p11.2 BP4-BP5 deletion

Our typical 16p11.2 BP4-BP5 deletion group consisted of 62 cases (median age 11.9 years). In the majority of cases, the deletion occurred de novo. One case had a typical 16p11.2 BP4-BP5 deletion that was reported de novo by initial testing in 2013 (Agilent 180 K custom HD-DGH microarray, UCSC Feb 2009, NCBI build 37.1/Hg19). The affected case, however, had a sibling with minor symptoms, who turned out to carry the same CNV. Repeated testing of the clinically unaffected mother, this time with SNP array, revealed that she carries the same deletion, yet in mosaic form. The vast majority of cases (85.5%) had DD, often with speech delay. Autistic features were common and 19.4% had a formal diagnosis of ASD. Part of the cases (*n* = 33) had an IQ test (mean FSIQ 84, ID in 18.2%). ADHD was seen in 27.3%, yet information on this was not available of all cases. Half of cases had high BMI (21.3% overweight, 29.5% obesity, average age of onset of obesity of 9.8 years) and 41% reported hyperphagia. Mean height SDS was -0.8 and mean BMI SDS was 1.5. OFC was typically normal (mean OFC SDS 0.5, 8.2% macrocephaly). Approximately one third of cases had sleep problems. Almost half of cases had vision problems, which included refractive errors (e.g. myopia). Epilepsy was seen in 9.7%, which is less frequent than previously reported [4, 26, 27].

#### Distal 16p11.2 BP2-BP3 deletion

After exclusion of case 18 (diagnosed with Noonan syndrome), our distal 16p11.2 BP2-BP3 deletion group consisted of 19 cases (median age 14.6 years). The distal 16p11.2 BP2-BP3 deletion was de novo in 25% of cases. Development, both motor and speech development, was typically delayed. Of the cases with available IQ scores (n = 10), 40% had ID (mean FSIQ 83). Autistic features were common and a diagnosis of ASD was made in 22.2%. The vast majority of cases had obesity (73.7%, mean age of onset 5.1 years) and hyperphagia (61.1%). Mean height was normal, yet BMI SDS was 3.2 and OFC SDS was 0.9. More than half of cases (63.2%) had vision problems. Additionally, more than half of cases (57.9%) had sleeping problems. Epilepsy was reported in 15.8%.

#### Typical 16p11.2 BP4-BP5 versus distal 16p11.2 BP2-BP3 deletion

The majority of the typical 16p11.2 BP4-BP5 deletions were reported as de novo, which significantly differed to the distal 16p11.2 BP2-BP3 deletions (respectively 74.5% versus 25% de novo, *p* = 0.018), yet not all parents were tested (see Table 3B). Obesity was more frequently associated with the distal 16p11.2 BP2-BP3 than typical 16p11.2 BP4-BP5 deletions (*p* = 0.001). Cases with distal 16p11.2 BP2-BP3 deletions on average had an earlier onset of obesity (5.1 years versus 9.8 years; *p* = 0.044), as well as a higher BMI SDS ( + 3.2 versus +1.5; *p* < 0.001). Cases with typical 16p11.2 BP4-BP5 deletion had a lower height SDS than cases with distal 16p11.2 BP2-BP3 deletion (-0.8 versus +0.1; *p* = 0.007). Delay in motor development was more often reported in the distal 16p11.2 BP2-BP3 deletion group (*p* = 0.010) and neonatal feeding problems more often reported in the typical 16p11.2 BP4-BP5 deletion group (p = 0.031).

#### Other 16p11.2 deletions

Three cases had other, larger, pathogenic 16p11.2 deletions (Fig. 1B and Table 1).

One case (8.3 year old male) had a de novo 28.3–30.2 Mb (size 1.9 Mb) 16p11.2 deletion. He was SGA (birthweight <10th centile), had DD (psychomotor and speech/language), normal IQ, frequent ear infections, overweight (BMI 21.5 kg/m²; +2.9 SDS) and hyperphagia. Height (136.5 cm; +0.3 SDS) and OFC (54.5 cm; +1.1 SDS) were normal. He had pes planovalgus.

Another case (9.9 year old male) had a de novo 28.4–30.2 Mb (size 1.8 Mb) 16p11.2 deletion. This case had DD (psychomotor and speech/language), borderline intellectual functioning (FSIQ 72), ASD, ADHD, urinary incontinence, premature pubarche, block vertebra L5/S1, with an open arch S1, previous obesity (currently overweight; BMI 21.1 kg/m², +2.3 SDS, age of onset 4 years) and signs of hyperphagia. Height (138.7 cm; -0.6 SDS) and OFC (52.5 cm; -0.4 SDS) were normal.

Finally, the third case (11.1 year old male) had a paternal 28.4–29.4 Mb (size 1 Mb) 16p11.2 deletion. He had a normal initial development, borderline intellectual functioning (FSIQ 76), no behavioral issues, obesity (BMI 26.2 kg/m²; +3.2 SDS, age of onset 6 years), no hyperphagia, cryptorchidism and headaches. Height (152.5 cm; +0.5 SDS) and OFC (53 cm; –0.4 SDS) were normal.

#### Typical 16p11.2 BP4-BP5 duplication

Our typical 16p11.2 BP4-BP5 duplication group consisted of 10 cases (median age 8.2 years). Neonatal feeding problems were present in 30% of cases. More than half of cases (60%) had DD. Of cases who had an IQ test (*n* = 7), 57.1% had ID and mean FSIQ was 73.7. Features of autism were frequently reported, yet only 10% had a formal diagnosis of ASD. A formal diagnosis of ADHD was made in 30% of cases. One case had obesity, while all other cases had a normal weight, with a mean BMI SDS of 0.1. Mean height SDS was -0.8. Two cases (20%) had microcephaly. The group had a mean OFC SDS of -1. Sleeping problems were common (60%), as well as constipation (30%) and vision problems (30%). Epilepsy was reported in one case (10%).

#### Distal 16p11.2 BP2-BP3 duplication

Only three cases had a distal 16p11.2 BP2-BP3 duplication (mean age 6.4 years). All had DD. One had ID. ASD and ADHD were not reported. One case had overweight, the other two cases had a normal weight. Mean height SDS was -0.8, mean BMI SDS was 0.6, mean OFC SDS was -1.9. Microcephaly was observed in two out of three cases.

#### Other 16p11.2 duplications

Two cases had other 16p11.2 duplications (Fig. 1B and Table 1).

One of them (girl, 12.4 years old) had a de novo 28.3–30.3 Mb (size 2 Mb) 16p11.2 duplication. She had polydactyly bilaterally. Initial development was normal, but learning problems were reported later in childhood. An IQ test showed a disharmonic profile (VIQ 90, NVIQ 60, FSIQ not known). ASD was formally diagnosed. She had sleeping problems for which she received melatonin (5 mg) with little to no effect. Feeding has always been difficult. Height (153 cm, –0.7 SDS), weight (BMI 17.1 kg/m², –0.3 SDS) and OFC (53 cm, –0.6 SDS) were normal. Epicanthic folds and long slim fingers with hypermobility were observed.

The second case (girl, 11.9 years old) had a de novo 21.5–30 Mb (size 8.5 Mb) 16p11.2 duplication. She had delayed development, ID, ASD, myopia (–1.5 diopters (D)/-2 D), congenital hip dysplasia and short stature (height 141 cm, –2.1 SDS). Furthermore, she had sleeping difficulties, for which melatonin treatment had good effect. Clinical examination showed a normal BMI (15 kg/m²; –1.4 SDS) and OFC (52 cm, –1 SDS). Full eyelids, epicanthic folds, subtle synophrys, three café-au-lait macules and hypertrichosis of legs were observed.

#### Medication use in total 16p11.2 CNV cohort

Half of the cases in our total 16p11.2 CNV cohort used a wide variety of prescribed drugs; psychotropic drugs (most often methylphenidate), anti-epileptics, sleep medication, medication for constipation, asthma, diabetes and less frequently for other disorders.

## Discussion

We here present the largest clinical Dutch cohort with different pathogenic 16p11.2 deletions and duplications. All 100 cases were assessed at our clinical genetics center (Amsterdam UMC). Genetic testing was indicated by clinical presentation. CMA is often a first-tier test for cases with ID and/or congenital anomalies [28]. It is likely that we have mainly seen cases with more severe phenotypes. We did however also include relatives (parents, siblings) that carried the same 16p11.2 CNV, who had questions about recurrence risks, instead of their own clinical phenotype. It is known that cases with 16p11.2 CNVs exhibit large clinical heterogeneity, as we see in our cohort. In general, the prevalence of clinical features in our cohort of cases with different 16p11.2 CNVs resembles previously described large cohorts (Fig. 3, Table 3A, 4).

Suspected genetic obesity can be an indication for genetic testing. Since we are an obesity genetics expertise center, our obesity gene panel includes a 16p11.2 CNV analysis. It is therefore not surprising that we have seen more cases with 16p11.2 deletions (associated with obesity) than cases with 16p11.2 duplications (associated with underweight). However, our typical 16p11.2 BP4-BP5 deletion subgroup itself had a lower overall prevalence of obesity (29.5%) than previously reported 50–75%, see Table 3A [3, 27]. This might be explained by the different indications for genetic analysis (e.g. DD/ID) and earlier age of testing. In our cohort, the majority (75.8%) of cases with typical 16p11.2 BP4-BP5 deletion was younger than 18 years and almost half (45.2%) 10 years or younger, while average age of obesity onset in this group was 10.2 years. Part of cases with typical 16p11.2 BP4-BP5 deletion in our cohort (21.3%) were classified as overweight. So, even though we currently found a lower than expected prevalence of obesity in this subgroup, our experience is that young cases could still develop obesity at an older age (>10 years). Longitudinal follow-up on weight is thus indicated and will provide more information. Our distal 16p11.2 BP2-BP3 deletion subgroup showed a more severe obesity phenotype (73.7% of cases, earlier age of onset and higher average BMI SDS) than cases with typical 16p11.2 BP4-BP5 deletion (Table 3A, B). An explanation for this is that the affected regions consist of different genes (Fig. 1 and Table 1). The *SH2B1* gene (OMIM #608937), located within the specific distal 16p11.2 BP2-BP3 (28.8–29.0 Mb) deletion region, has in particular been linked to obesity, through effects on leptin and insulin signaling [29]. Since setmelanotide, a melanocortin-4-receptor (MC4R) agonist, is being evaluated as anti-obesity treatment for certain cases with monogenic obesity [30], including cases with an *SH2B1* variant, our cases with distal 16p11.2 BP2-BP3 deletions are currently eligible for these clinical trials. Pharmaceutical anti-obesity treatment options for all 16p11.2 deletion subgroups are desired, as cases with typical 16p11.2 BP4-BP5 deletion are also prone to develop obesity [27, 31]. Apart from early genetic diagnosis, combined lifestyle and dietary interventions, a glucagon-like peptide 1 (GLP-1) analog, liraglutide, was recently shown to be effective as anti-obesity treatment for one case with typical and one case with distal 16p11.2 BP2-BP3 deletion [32]. More knowledge of 16p11.2 syndromes and a personalized approach for neurocognitive and environmental factors and medication use can hopefully reduce or prevent obesity in future cases with 16p11.2 deletions. In our cohort, two cases with typical 16p11.2 BP4-BP5 deletion were underweight. One of them was born preterm and SGA, had a short stature (height –3.2 SDS at age 2 years), sacral dimple, bicuspid aortic valve and VSD. The other case with typical 16p11.2 BP4-BP5 deletion and underweight was also born SGA and had a short stature (height –2.7 SDS at age 7). Extensive diagnostics did not reveal an additional genetic diagnosis yet. On the contrary, one case with a typical 16p11.2 BP4-BP5 duplication had slight obesity (adult BMI 31.5 kg/m², age of onset unknown, no hyperphagia). Surprisingly, none of our cases with a 16p11.2 duplication were underweight at time of assessment. However, significantly lower BMI SDS and also OFC SDS were seen in the 16p11.2 duplication groups, compared to the 16p11.2 deletion groups (Fig. 3).

In current clinical genetic practice, 5–7% of cases with ID/congenital malformations are diagnosed with two different Mendelian disorders [33, 34]. Dual diagnoses have also been described for 16p11.2 deletion syndrome [35, 36]. Trained clinicians will have to decide whether more extensive genetic testing, following a molecular 16p11.2 deletion or duplication diagnosis, is recommended. In our cohort, additional genetic testing was performed in the majority of cases. Dual diagnoses identified in our cohort, should encourage to pay close attention to whether an individual’s phenotype is fully explained by the 16p11.2 CNV (Table 2). For example, one case with distal 16p11.2 BP2-BP3 deletion had a short stature (height (SDS –2.9)) and congenital heart defect, both cardinal features of Noonan syndrome. We indeed confirmed this second diagnosis. As this second diagnosis could affect our results and conclusions, we excluded this particular case from our subgroup analyses. Clinical overlap of two syndromes makes it harder to realize that these cases could have a dual diagnosis [36]. We recommend consultation of a clinical geneticist in case of severe or previously unreported symptoms. Importantly, we mentioned one case with an apparent de novo typical 16p11.2 BP4-BP5 deletion when tested using array-CGH, whose mother eventually carried the same typical 16p11.2 BP4-BP5 deletion in mosaic form when reanalyzed with SNP array nine years later. Even though this is a rare finding, it can be relevant for siblings of cases with an apparent de novo deletion, who were analyzed with previous genetic techniques.

Our findings of high percentages of DD in all subgroups and a more often reported delayed speech development in cases with typical 16p11.2 BP4-BP5 and distal 16p11.2 BP2-BP3 deletions, was similar to previous reports [3, 9, 18, 37, 38]. Cases with more severe learning difficulties are most likely to undergo an IQ test and some cases were too young for an IQ test. Of the cases with a 16p11.2 CNV and available FSIQ data, ID was reported in 28.6% and borderline intellectual functioning in 62.5%, similar to previously reported large (N ≥ 100 cases) studies [4, 39]. In our cohort, 18.2% of cases with typical 16p11.2 BP4-BP5 deletion were diagnosed with ID, similar to the reported 20-30% of cases with typical 16p11.2 BP4-BP5 deletion in previous large studies [4, 39]. Of cases with distal 16p11.2 BP2-BP3 deletion in our cohort, 40% were diagnosed with ID, yet FSIQ information was not available for approximately half of cases. Information about the prevalence of ID in cases with distal 16p11.2 BP2-BP3 deletions is not available in current literature, however the Simons Searchlight registry [40] reports a combined prevalence of DD or ID in 52% of cases with distal 16p11.2 BP2-BP3 deletion. A formal diagnosis of ASD was made in 19.4% of cases with typical 16p11.2 BP4-BP5 deletion in our cohort, compared to 11-45% of cases in different large cohorts [4, 27, 39, 40]. Another 35.5% of cases with typical 16p11.2 BP4-BP5 deletion in our cohort had signs of ASD without formal ASD diagnosis. Cases with typical 16p11.2 BP4-BP5 duplications in our cohort had a higher prevalence of DD (60%) and ID (57.1%) than reported in larger cohorts in literature, where DD was reported in 48% [40] and ID in 30.5% [4] of cases. However, our subgroup of cases with typical 16p11.2 BP4-BP5 consists of only 10 cases, compared to larger cohorts of 142 [40] and 270 [4] cases. ASD, on the other hand, was officially diagnosed in only 10% of cases in our cohort, compared to 40% in the Simons Searchlight registry [40]. An explanation for this might be that an additional 50% of cases in our cohort had features of ASD but were not officially diagnosed with ASD.

Information about adaptive functioning and disharmonic intelligence profiles was incomplete and not taken into account in our study. The variety of neurocognitive problems associated with pathogenic 16p11.2 CNVs [39] can lead to difficulties in daily functioning. Early diagnosis and guidelines for support and treatment are important for overall functioning and quality of life. For all cases with a 16p11.2 CNV and DD/ID, early intervention services, such as speech or physical therapy and educational interventions may be helpful. We advise to perform an IQ assessment at least once and more often (particularly at every transition between educational levels) when learning problems are observed. Some cases can benefit from psychological/psychiatric evaluation and interventions to address behavioral and emotional problems, such as psycho-education or cognitive-behavioral therapy. Clinicians should be careful with prescribing potentially obesogenic psychotropic medication for cases with 16p11.2 deletion and anorexic medication to cases with 16p11.2 duplication. A pharmacogenetic passport [41] will be offered to cases with 16p11.2 CNV who visit our clinics. Such a passport provides recommendations for medication choice and dosage, based on an individuals’ genotype data, to minimize side-effects and optimize personalized medical treatment. An overview of our clinical recommendations is provided in Supplementary Table S2.

The observed clinical heterogeneity in cases with 16p11.2 CNV may be the result of a combination of additional (epi)genetic and/or environmental factors. Our future research will focus on exploring this by assessing genome-wide DNA methylation data and the contribution of additional genetic factors (using ‘polygenic risk scores (PRS)’). Certain genetic defects can lead to disorder-specific genome-wide DNA methylation patterns, so-called ‘episignatures’. These episignatures are rapidly being developed and can, apart from facilitating earlier diagnoses, also help in understanding the underlying pathophysiological mechanisms [42]. An episignature for the typical 16p11.2 BP4-BP5 deletion has already been described [43]. We are currently further investigating if episignatures for the different 16p11.2 CNVs can be distinguished. We are also exploring whether specific phenotypes within these subgroups can be explained by particular differences in DNA methylation, in some cases perhaps because of a second diagnosis. Furthermore, we are investigating the contribution of common genetic variants (SNPs), observed in genome-wide association studies (GWAS) for certain complex traits (e.g. BMI, height), to the clinical variability observed in cases with 16p11.2 CNVs. A single GWAS SNP may only slightly affect an individuals’ phenotype, but particular combinations of multiple SNPs can significantly alter clinical outcomes. PRS captures the combined effects of these GWAS variants and can be calculated for various clinical traits of interest [44]. Previous studies that assessed the clinical relevance of additional other (rare and common) genetic variants showed a higher burden of other rare genetic variants in cases with a 16p11.2 deletion and ID compared to cases without ID [45], but also that a high PRS can increase the risk of developing schizophrenia for cases with particular CNVs [46, 47]. Interestingly, a higher burden of rare genetic variants could also affect other features, like OFC SDS [45], which could be an explanation for a lower average OFC SDS and lower prevalence of macrocephaly in our typical 16p11.2 BP4-BP5 deletion group (Fig. 3, Table 3A) compared to previously described cohorts [4, 26, 27].

In conclusion, we here present the clinical manifestations of our Dutch cohort of 100 cases with different pathogenic 16p11.2 CNVs. As the phenotype is complex and variable, we emphasize the importance of a personalized and multidisciplinary approach for all cases, with an emphasis on cognitive, psychiatric, growth parameters evaluation and treatment. Additional genetic diagnostics can be considered by trained clinicians, to explain uncommon features. Polygenic and epigenetic assessment will further predict clinical variability and personalized treatment in the future.

## Supplementary information

Supplementary tables

## Data Availability

Data is presented in figures and tables in the text and in supplemental files.
