# Supplementary material for: Evaluation of 100 Dutch cases with 16p11.2 deletion and duplication syndromes; from clinical manifestations towards personalized treatment options
Source: Eur J Hum Genet. 2024 Apr 11;32(11):1387–401. doi: 10.1038/s41431-024-01601-2 (PMC11576736; doi:10.1038/s41431-024-01601-2)
Supplement: Supplementary file 1 — Supplementary tables [file 41431_2024_1601_MOESM1_ESM.pdf]

S1 Supplementary table – Other findings

Overview of less commonly reported clinical features in the 16p11.2 CNV groups.

	**Total 16p11.2 deletion group**	**Total 16p11.2 duplication group**
Psychiatric	tics (n=2)<br>alcohol abuse (n=2)<br>schizophrenia (n=1)<br>auditory hallucinations (n=1)	anger/aggression (n=5)<br>impulsivity (n=2)<br>depression (n=2)<br>anxiety (n=1)<br>tics (n=1)
Urogenital	cryptorchidism (n=2)<br>unilateral renal agenesis (n=2)<br>bladder polyp (n=1)	-
Cardiovascular	atrial septal defect (n=2)<br>pulmonary valve stenosis (n=2)<br>arrhythmia (Wolff-Parkinson-White syndrome (WPW); n=2)<br>ventricular septum defect (n=1)<br>persistent ductus arteriosus (n=1)<br>bicuspid aortic valve (n=1)<br>narrow aorta (n=1)<br>undefined heart murmur (n=1)	-
Other	velopharyngeal insufficiency (n=2)<br>tendinitis (n=2)<br>inguinal hernia (n=2)<br>obstructive sleep apnea (n=2)<br>oculocutaneous albinism (n=1)<br>hippocampal dysplasia (n=1)<br>hypertrophic pyloric stenosis (n=1)<br>perniones (n=1)<br>immune thrombocytopenia (ITP; n=1)<br>frequent spontaneous epistaxis (n=1)<br>Arnold Chiari malformation 1 (n=1)<br>hidradenitis (n=1)<br>bilateral thumb hypoplasia (n=1)<br>ear canal stenosis (n=1)<br>hyperandrogenism (n=1)<br>Perthes disease (n=1)<br>multiple fractures (n=1)<br>laryngomalacia (n=1)<br>external hydrocephalus (n=1)<br>mitochondrial respiratory chain disorder (n=1)<br>brain tumor (n=1)<br>non-alcoholic steatohepatitis (NASH; n=1)<br>keratosis pilaris (n=1)<br>neck fistula (n=1)<br>collarbone fistula (n=1)<br>ear fistula (n=1)	lateral neck cyst (n=2)<br>primary amenorrhea (n=2)<br>inguinal hernia (n=2)<br>hip dysplasia (n=1)<br>anal atresia (n=1)<br>bladder dysfunction (n=1)<br>hypospadia (n=1)<br>amblyopia (n=1)

S2 Supplementary table – Clinical recommendations after 16p11.2 CNV diagnosis

Based on GeneReviews, Unique disorder guides, and this publication.

**All cases with 16p11.2 CNV**	
Monitor early development	- Refer for speech therapy and/or physical therapy if speech and/or motor difficulties become apparent.
IQ assessment	- Assess IQ at least once to estimate learning capacities and organize additional learning support if required.<br>- Repeat IQ assessment if needed, when learning difficulties become apparent and in particular when changing schools.<br>- In case of ID, patients can be referred to an ID physician.
Monitor growth parameters	- Monitor height and weight annually during childhood as patients can be prone to underweight (16p11.2 duplication) or overweight/obesity (16p11.2 deletion).<br>- Monitoring preferably by pediatric endocrinologist, as further endocrine assessment can be indicated.<br>- Refer to a dietician in case of severe underweight or obesity (any age).
Consult clinical geneticist	- Counseling by clinical geneticist for general information and explanation of recurrence risk.<br>- Including dysmorphology evaluation for assessment of potential second genetic diagnosis.<br>- Discuss personalized medicine options and ongoing research programs at expertise center.
Medication evaluation	- Psychotropic and other medication are frequently prescribed. Consider pharmacogenetic passport for all 16p11.2 CNV cases to reduce side effects and optimize personalized therapy.<br>- Preferably prescribe non-obesogenic medication to 16p11.2 deletion cases. Preferably do not prescribe anorexic medication to patients with 16p11.2 duplication, particularly with underweight.
**On indication, if particular symptoms arise**	
Psychological/psychiatric evaluation	- Recommended when remarkable behavior is observed or psychiatric symptoms are reported (ADHD, ASD and/or other behavioral and psychiatric symptoms).
Neurological evaluation	- If neurological symptoms arise, neurological evaluation and additional diagnostic techniques (EEG, MRI) can be considered.
Imaging of spine, heart and urogenital tract	- Perform on indication. Cases with 16p11.2 CNV are prone to (congenital) anomalies of these organs.
